# Evaluation of *MMP2* as a candidate gene for high myopia

**Published:** 2013-01-28

**Authors:** Bo Gong, Xiaoqi Liu, Dingding Zhang, Pu Wang, Lulin Huang, Ying Lin, Fang Lu, Shi Ma, Jing Cheng, Rong Chen, Xiaobo Li, He Lin, Guangqun Zeng, Xiong Zhu, Jianbin Hu, Zhenglin Yang, Yi Shi

**Affiliations:** 1 Sichuan Key Laboratory for Disease Gene Study, Sichuan Academy of Medical Sciences & Sichuan Provincial People’s Hospital, Chengdu, Sichuan, China; 2 Department of Laboratory Medicine, Sichuan Academy of Medical Sciences & Sichuan Provincial People’s Hospital, Chengdu, Sichuan, China; 3 Chengdu Institute of Biology, Chinese Academy of Sciences, Chengdu, Sichuan, China; 4 School of Materials Science and Engineering, Southwest Jiaotong University, Chengdu, Sichuan, China; 5 Department of Ophthalmology, Sichuan Academy of Medical Sciences & Sichuan Provincial People’s Hospital, Chengdu, Sichuan, China

## Abstract

**Purpose:**

Matrix metalloproteinase 2 (MMP2) has been shown to be expressed in the human sclera, and is increased in the sclera of the eye with myopia induced by form deprivation in chicks when compared with the control eye. The purpose of this study was to examine the relationship between high myopia and *MMP2* in a mainland Han Chinese population.

**Methods:**

Four hundred unrelated patients with high myopia and 400 normal controls in a mainland Han Chinese population were studied. All the subjects were genotyped for 20 tag single nucleotide polymorphisms (SNPs) in *MMP2* with the dye terminator-based SNaPshot method. The distribution of the genotypes in the cases and controls was compared with a χ^2^ test. Screening for mutations in the coding regions and the adjacent intronic regions of *MMP2* was performed in 200 patients with high myopia and 200 normal controls by direct sequencing.

**Results:**

None of the 20 tested SNPs showed significant association with high myopia in this study. Seven variations were detected upon sequencing of the coding regions and the adjacent intronic regions of *MMP2* in 200 subjects with high myopia and 200 normal controls. One novel variation, c.1287G>A (p.K429K), was detected in 79 of the 200 patients with high myopia (65 heterozygous and 14 homozygous) and in 84 of the 200 controls (67 heterozygous and 17 homozygous). The c.1810G>A mutation (p. Arg500His) was detected in three of the 200 patients with high myopia but not in the controls. The five other variations, known as polymorphisms, were detected in the case and control groups.

**Conclusions:**

We found no evidence that *MMP2* is responsible for high myopia in these Han Chinese subjects and hence is unlikely to be important in the genetic predisposition to high myopia. Our results imply that *MMP2* may not play a major role in high myopia in the Han Chinese population.

## Introduction

Myopia, the most common eye disease worldwide, is also the leading cause of visual impairment. Myopia can be classified as low, medium, or high myopia. High myopia is defined as having a spherical equivalent of less than or equal to −6.00 diopter sphere (DS) and an axial length longer than or equal to 26.0 mm [1]. High myopia is a complex disease associated with environmental and genetic factors. Environmental factors such as work at close range and prolonged reading are suggested to be involved in the progression of myopia [2]. Family studies have shown an increased risk of myopia in children with myopic parents, compared with those with no myopic parents [3], as well as a fourfold increased sibling risk [4]. However, although abundant evidence has demonstrated that genetic factors play an important role in the development of high myopia, the exact molecular basis of high myopia and the genes that cause a predisposition to this disorder are still unclear.

Matrix metalloproteinases (MMPs) are a family of zinc-dependent endopeptidases that degrade extracellular matrix proteins; more than 20 members of the MMP family have been identified in humans [5]. Among them, MMP-1[6], -2[6,7], -3[6], and -14[7] have been shown to be expressed in the human sclera and are potential participants in scleral remodeling. MMP2 is increased in the sclera of the eye with myopia induced by form deprivation in chicks when compared with the control eye [8-10]. Increased scleral MMP2 expression in form-deprivation myopia has been shown in tree shrews at the protein [11] and the messenger RNA (mRNA) levels [12,13] and in guinea pigs at the protein level [14]. An increased MMP2 transcript level has also been found in human scleral ﬁbroblasts mechanically stretched in an in vitro system [15] and in lens-induced myopia in the tree shrew [16]. For high myopia, variations in the expression of the *MMP* genes in the sclera due to polymorphisms in the promoter regions can cause variations in scleral remodeling activity, a key factor in axial elongation of the eye. The expression of many MMPs is regulated mainly at the transcription level, and SNPs in the promoter region of several *MMP* genes have been shown to be transcriptional regulators [17]. *MMP2* has been shown to have functional SNPs in the promoter regions, such as rs243865 (*MMP2* C-1306T) [18] and rs2285053 (*MMP2* C-735T) [19]. In an Amish population, rs9928731 showed evidence of association with refractive phenotypes, located between the sixth and seventh exons of *MMP2* [20]. However, no signiﬁcant difference was detected in the distribution of the two SNPs (rs243865 and rs2285053) and the other 17 SNPs in *MMP2* between high myopia cases and general-population controls in a Japanese population [21] and in a Hong Kong Han Chinese****population [22], respectively.

In this study, thus, we sought to evaluate *MMP2* as a candidate gene for high myopia. We examined the relationship between high myopia in a mainland Han Chinese population composed of 400 subjects with high myopia and 400 normal controls. All the coding regions were sequenced to screen novel variants in *MMP2*.

## Methods

### Subjects

In total, 400 patients with high myopia and 400 matched normal controls were recruited from Sichuan Academy of Medical Sciences & Sichuan Provincial People’s Hospital. Clinical information about the cases and controls is listed in Table 1. This study was approved by the Institutional Review Boards of the Sichuan Academy of Medical Sciences & Sichuan Provincial People’s Hospital. Written informed consent was obtained from all subjects before the studies, and the subjects underwent an extensive, standardized examination by ophthalmologists, including visual acuity testing, a detailed clinical examination, optical coherence tomography, and ocular imaging before genetic testing. Refractive error and the radius of corneal curvature in the horizontal and vertical meridian were measured using an autorefractor (KR8800, Topcon, Tokyo, Japan). Subjects with syndromic disorders or systemic diseases that could lead to myopia were excluded. High myopia is defined by a spherical equivalent of less than or equal to −6.00 diopter sphere (DS) and an axial length longer than or equal to 26.0 mm in affected patients’ eyes. For the controls, the criteria were a spherical equivalent from –1.0 to +1.0 DS, an axial length less than or equal to 24.0 mm, and no evidence of disease in either eye.

**Table 1:** Characteristics of high myopia cases and controls in this study.

Groups	Number	Age (Years)*	Refractive errors (Diopter, ±)	Axial length (mm)
Cases	400	33.3±10.7	−9.97±3.14 (OD), −9.79±3.26 (OS)	27.56±1.85 (OD), 27.85±1.78 (OS)
Controls	400	51.1±9.5	−0.41±0.56 (OD),	23.37±0.71 (OD),
			−0.43±0.59 (OS)	23.42±0.75 (OS)

*The age when the cases and controls were recruited. ±: standard deviation; OD: right eye; OS: left eye; mm: millimeter.

### Single nucleotide polymorphism selection and genotyping

We selected 20 tag SNPs, including rs243865, rs2285053, and so on in *MMP2*, to be genotyped in the mainland Han Chinese population (400 patients with high myopia and 400 normal controls). Venous blood was drawn from cubital veins of each subject and collected in an EDTA tube. The blood samples were preserved at -80 °C before genomic DNA extraction. Genomic DNA was extracted from the blood by serial phenol/chloroform extraction and ethanol precipitation. SNP genotyping was performed with the dye terminator-based SNaPshot method (Applied Biosystems, Foster City, CA). SNP analysis was performed on the ABI 3130 Genetic Analyzer (Applied Biosystems). In brief, the PCRs (10 μl ﬁnal volume) contained 50 ng of genomic DNA, 1 μl of each primer (10 pmol/μl), 1 μl of 10 buffer (Takara Bio Inc., Shiga, Japan), 0.8 μl of deoxyribonucleotide triphosphates (2 mmol/l; Takara Bio Inc.), 0.4 μl MgCl_2_ (2.5 mmol/l; Takara Bio Inc.), and 0.1 μl of ExTaq polymerase (5 U/μl; Takara Bio Inc.). The product was then processed according to the ABI SNaPshot protocol.

### Mutation analysis

Screening for mutations in *MMP2* was initially performed in 200 patients with high myopia and 200 matched normal controls. Amplified PCR products of all the coding exons and adjacent introns (the sequences of all primers used in this study are summarized in Table 2) were purified with spin columns (QIAquick, Qiagen, Valencia, CA) and sequenced directly (BigDye Terminators Sequencing Kit; Applied Biosytems) in both directions with an automated genetic analysis system (3130; ABI).

**Table 2:** Primers used for mutation screening in *MMP*2 gene.

Exon	Primer sequence(5′-3′)	Product size (bp)	Annealing temperature (°C)
1	F: GTACTGTGCCATCCTAAT	445	56
	R: CTGTCTGACTTCATTTTCT		
2	F: CACATACACGCAGGCACA	614	62
	R: CCATATTGGACAGCACAGT		
3 and 4	F: TTTCAGGGTCTAGGTGGC	677	62
	R: GGAACTGAGTGAAGGACG		
5	F: GAGAAGCAGCTCCTTACCA	463	59
	R: GGATGTCATTCGCACAGAT		
6	F: AGCGTCATGTCATTGCTT	378	62
	R: CTGGGTAGGTGGGTGTCT		
7	F: ACAAGAAGACTTTGGCTGAC	595	59
	R: TTCGGATAGGGAAGAGTTA		
8	F: AGAGGACTGATTTGGGTGAT	404	62
	R: GGACAGGAGACAAGGAGG		
9	F: CAGGGTAGGAGGATGTTTC	561	61
	R: AATGCTATCTGATGTTGGGT		
10	F: TGACTTCTAAAGCCCTCTG	375	59
	R: AACTGTGCTGCTGTCCTAC		
11	F: AAGGGCTAGGTCCAGTTTC	414	59
	R: CAAGGAGCAGAGGTCAGG		
12	F: TGGGCTCAAGCAATCCTC	309	59
	R: TGTATCGAAGGCAGTGGA		

### Statistical analysis

Hardy–Weinberg equilibrium (HWE) for each SNP polymorphism was tested with the χ^2^ test. P values of the SNPs were calculated using an additive model. The unadjusted odds ratios (ORs) of the alleles and genotypes between the cases and controls were estimated with the χ^2^ test. All statistical analyses were performed using the software SPSS 15.0 (SPSS Inc., Chicago, IL). Genetic power was calculated by using the software PS: Power and Sample Size Calculation (PS version 3.0.43) [23].

## Results

### Clinical data

Eight hundred unrelated subjects were included in the study. The cohort consisted of 400 cases and 400 controls. For subjects with high myopia, the spherical refractive errors of the right and left eyes were –9.97±3.14 D and –9.79±3.26 D, respectively; the axial lengths of the right and left eyes were 27.56±1.85 mm and 27.85±1.78 mm, respectively (Table 1).

### Single nucleotide polymorphism analysis

In total, 20 SNPs were genotyped in HWE (p>0.05) for *MMP2*, including 16 in intronic regions, one in exon 4, and three upstream of the 5′ region (Table 3). After association analysis of these SNPs, none of the 20 tested SNPs showed significant association with high myopia in the mainland Han Chinese population (400 patients with high myopia and 400 matched normal controls) (Table 3). In the previous study [20], rs9928731 in the *MMP2* gene showed evidence of association with refractive phenotypes (p=0.00026) in Amish families. Therefore, we calculated the power of rs9928731 for detecting moderate/low ORs in the range of 1.2–1.8 based on our sample size. The power values ranged from 42.4% to 100% (42.4%, 72.0%, 90.4%, 97.60%, 99.50%, 99.90%, and 100.00% for OR=1.2, 1.3, 1.4, 1.5, 1.6, 1.7, and 1.8, respectively). The data suggested sufficient power to reject the null hypothesis of no association between rs9928731 and high myopia. Thus, the genotyping results indicated that there were no significant differences in the SNPs between the patients with high myopia and the controls.

**Table 3:** SNP genotyping of the *MMP2* gene in 400 high myopia and 400 control subjects

SNPs	Position (bp)	Allele*	Frequency of reference allele		P value	OR (95% CI)
			Cases	Controls		
rs11643630	54,067,960	G/T	0.473	0.48	0.763	0.97 (0.80–1.18)
rs243865	54,069,307	C/T	0.869	0.879	0.547	0.91 (0.70–1.23)
rs2285053	54,069,878	C/T	0.704	0.681	0.329	1.11 (0.90–1.37)
rs1477017	54,074,663	G/A	0.275	0.3	0.269	0.89 (0.71–1.10)
rs865094	54,074,733	G/A	0.323	0.334	0.632	0.95 (0.77–1.17)
rs11076101	54,075,759	C/T	0.866	0.86	0.716	1.05 (0.79–1.40)
rs17301608	54,076,111	C/T	0.644	0.664	0.4	0.92 (0.74–1.12)
rs11646643	54,076,378	G/A	0.131	0.116	0.362	1.15 (0.85–1.55)
rs1132896	54,077,036	G/C	0.864	0.879	0.37	0.87 (0.65–1.17)
rs2241146	54,079,735	G/A	0.778	0.773	0.811	1.03 (0.82–1.30)
rs9928731	54,080,512	C/T	0.574	0.604	0.223	0.88 (0.72–1.08)
rs12599775	54,081,283	C/G	0.13	0.119	0.495	1.11 (0.82–1.49)
rs243847	54,081,499	C/T	0.416	0.409	0.76	1.03 (0.84–1.26)
rs243845	54,083,988	G/A	0.709	0.684	0.277	1.12 (0.90–1.39)
rs243843	54,084,799	G/A	0.431	0.449	0.481	0.93 (0.76–1.13)
rs183112	54,085,183	G/A	0.734	0.756	0.302	0.89 (0.71–1.11)
rs1992116	54,085,392	G/A	0.711	0.734	0.315	0.89 (0.72–1.11)
rs11639960	54,090,771	G/A	0.28	0.268	0.575	1.23 (0.98–1.54)
rs243835	54,094,123	C/T	0.389	0.378	0.643	1.04 (0.86–1.28)
rs1861320	54,098,541	G/T	0.74	0.765	0.247	0.87 (0.70–1.10)

* The alleles are named with reference to the sense/anti-sense strand of the respective gene.

### Mutation analysis

Complete sequencing of the coding regions and the adjacent intronic regions of *MMP2* in the 200 subjects with high myopia and 200 normal controls identified seven variations (Table 4). One novel variation, c.1287G>A (p.K429K), was detected in 79 of the 200 patients with high myopia (65 heterozygous and 14 homozygous) and in 84 of the 200 controls (67 heterozygous and 17 homozygous). This variation would not affect the encoded amino acid. The six other variations were known polymorphisms, including three missense variations (c.1026T>C, c.1810G>A, and c.2172G>C) and three synonymous variations (c.1460T>C, c.1691G>C, and c.2117C>T; Table 4). All except c.1810G>A (rs28730814) were detected in the patients with high myopia and the control groups. The c.1810G>A mutation (p. Arg500His) was detected in only three of the 200 patients with high myopia (three heterozygous), but not in the 200 controls. In this heterozygous variation, an arginine is replaced by a histidine in the encoded protein. No variation was identified in exons 1, 2, 3, 5, 10, and 12 of *MMP2*.

**Table 4:** *MMP2* Variants detected in 200 high myopia and 200 control subjects by direct sequencing of all the exons *Minor allele/major allele.

Location	Position (bp)	SNP ID	Allele*	Residue Change	Genotype Counts †		Allelic p
					Cases	Controls	
Exon 4	54,077,073	rs11542001	T/C	F239L	0/1/199	0/3/197	0.32
Exon 6	54,081,206	rs243849	T/C	D383D	72/44/84	67/42/91	0.39
Exon 7	54,083,320	novel	G/A	K429K	14/65/121	17/67/116	0.51
Exon 8	54,084,614	rs2287074	G/A	T460T	32/67/101	28/60/112	0.25
Exon 9	54,088,365	rs28730814	G/A	R500H	0/3/197	0/0/200	-
Exon 11	54,094,228	rs10775332	C/T	F602F	13/60/127	11/63/126	0.93
Exon 11	54,094,283	rs16955280	G/C	V621L	0/2/198	0/1/199	0.56

†The genotype counts are presented as homozygote/heterozygote/wild-type.

## Discussion

Identifying the genes responsible for non-syndromic high myopia is very important but will be very difficult, although several loci for high myopia have been mapped [24-43]. However, no convincing causal genes have yet been identified at these loci. Differential MMP2 expression has been implicated in scleral remodeling in experimental myopia studies in tree shrews [11-13,16] and chicks [8]. In these form-deprivation animal models, myopic eyes show increased *MMP2* mRNA expression compared with that in normally developing eyes, leading to increased collagen degradation and active sclera remodeling. A similar mechanism may be involved in common forms of heritable human refractive error. In this study, thus, we sought to determine whether *MMP2* is associated with high myopia in a mainland Han Chinese population. First, we used a case-control study approach to examine the relationship between high myopia and the tag SNPs of *MMP2* in a mainland Han Chinese population. Then all the coding regions were sequenced to screen novel variants in *MMP2*.

In this study, 20 SNPs were genotyped for *MMP2*, and none showed significant association with high myopia in the mainland Han Chinese population. The known variation, rs9928731, was previously reported to be associated with Amish patients with high myopia [20], suggesting that this variation is more likely to be a susceptibility polymorphism of high myopia. However, this variation was not replicated in our study. When the coding regions and the adjacent intronic regions of *MMP2* in the 200 subjects with high myopia and the 200 normal controls were completely sequenced, one novel variation and six known variations were detected. All except c.1810G>A (rs28730814) were found in the patients with high myopia and the control groups.

Therefore, it is impossible to confirm or deny the susceptibility of the *MMP2* gene with high myopia based on the current evidence, especially because of our limited understanding of complex diseases. To our knowledge, several studies have been conducted to investigate the association of *MMP2* polymorphisms and refractive error phenotypes [18-22]. Nakanishi [21] detected no signiﬁcant difference in the distribution of two SNPs (rs243865 and rs2285053) between high myopia cases and general-population controls. The researchers did not find statistically significant associations with these SNPs in a full analysis of 216 cases and 474 controls. A second study, which comprised 55 Amish and 63 Ashkenazi Jewish families including 358 Amish and 535 Ashkenazi Jewish subjects, analyzed four tag SNPs of *MMP2* [20]. The study showed one SNP (rs9928731) was statistically associated with refractive phenotypes in the Amish subjects but not in the Ashkenazi Jewish subjects. The results suggested that the *MMP2* gene was involved in refractive variation in the Amish population. Finally, a separate case-controlled study composed of 656 patients with high myopia and 654 controls demonstrated that there was no significant association with the 17 polymorphisms of *MMP2* and high myopia in Southern Chinese subjects in Hong Kong [22]. Taken together, genetic and/or environmental heterogeneity most likely contributes to these differences in association results between ethnic groups.

As the weak linkage disequilibrium (LD) between common tag SNPs and rare casual variants, this indirect approach has low power in detecting association with rare variants. Rare variants could be identified by sequencing good candidate genes or even the whole genome for a very large number of samples [44]. However, when the role of the rare variants in high myopia was explored with DNA sequence analysis for the exons of *MMP2* in small numbers of patients with high myopia, no fruitful results were found. We detected seven variations. The genotyping of these variations except c.1810G>A (rs28730814) were similar between the patients and controls (Table 4), suggesting that these variations are more likely to be polymorphisms and implying that this gene does not carry common sequence variants that are capable of influencing its function and/or regulation in the relevant ocular tissue. Additional studies for mutation screening are necessary to evaluate the role of the *MMP2* gene in the genetic susceptibility to high myopia. In addition, the contribution of behavioral and environmental effects on high myopia should be considered.

In conclusion, we genotyped 20 SNPs at the *MMP2* gene in a Han Chinese group composed of 400 patients with high myopia and 400 controls. None of the SNPs showed significant association with high myopia (p>0.05), and no novel variation causing high myopia was detected with direct sequencing in *MMP2*. Our results thus failed to identify *MMP2* as a significant risk factor for high myopia in a mainland Han Chinese population. Therefore, the role of *MMP2* in controlling refractive development requires further study and refinement in animal models and human genetic epidemiologic studies.

## References

[1] Jacobi FK, Zrenner E, Broghammer M, Pusch CM (2005). A genetic perspective on myopia.. Cell Mol Life Sci.

[2] Saw SM (2003). A synopsis of the prevalence rates and environmental risk factors for myopia.. Clin Exp Optom.

[3] Liang CL, Yen E, Su JY, Liu C, Chang TY, Park N, Wu MJ, Lee S, Flynn JT, Juo SH (2004). Impact of family history of high myopia on level and onset of myopia.. Invest Ophthalmol Vis Sci.

[4] Lee KE, Klein BE, Klein R, Fine JP (2001). Aggregation of refractive error and 5-year changes in refractive error among families in the Beaver Dam Eye Study.. Arch Ophthalmol.

[5] Murphy G, Nagase H (2008). Progress in matrix metalloproteinase research.. Mol Aspects Med.

[6] Kim JW, Lindsey JD, Wang N, Weinreb RN (2001). Increased human scleral permeability with prostaglandin exposure.. Invest Ophthalmol Vis Sci.

[7] Shelton L, Rada JS (2007). Effects of cyclic mechanical stretch on extracellular matrix synthesis by human scleral fibroblasts.. Exp Eye Res.

[8] Rada JA, Brenza HL (1995). Increased latent gelatinase activity in the sclera of visually deprived chicks.. Invest Ophthalmol Vis Sci.

[9] Jones BE, Thompson EW, Hodos W, Waldbillig RJ, Chader GJ (1996). Scleral matrix metalloproteinases, serine proteinase activity and hydrational capacity are increased in myopia induced by retinal image degradation.. Exp Eye Res.

[10] Rada JA, Perry CA, Slover ML, Achen VR (1999). Gelatinase A and TIMP-2 expression in the fibrous sclera of myopic and recovering chick eyes.. Invest Ophthalmol Vis Sci.

[11] Guggenheim JA, McBrien NA (1996). Form-deprivation myopia induces activation of scleral matrix metalloproteinase-2 in tree shrew.. Invest Ophthalmol Vis Sci.

[12] Siegwart JT, Norton TT (2001). Steady state mRNA levels in tree shrew sclera with form-deprivation myopia and during recovery.. Invest Ophthalmol Vis Sci.

[13] Siegwart JT, Norton TT (2002). The time course of changes in mRNA levels in tree shrew sclera during induced myopia and recovery.. Invest Ophthalmol Vis Sci.

[14] Yang SR, Ye JJ, Long Q (2010). Expressions of collagen, matrix metalloproteases-2, and tissue inhibitor of matrix metalloproteinase-2 in the posterior sclera of newborn guinea pigs with negative lens-defocused myopia.. Zhongguo Yi Xue Ke Xue Yuan Xue Bao.

[15] Cui W, Bryant MR, Sweet PM, McDonnell PJ (2004). Changes in gene expression in response to mechanical strain in human scleral fibroblasts.. Exp Eye Res.

[16] Siegwart JT, Norton TT (2005). Selective regulation of MMP and TIMP mRNA levels in tree shrew sclera during minus lens compensation and recovery.. Invest Ophthalmol Vis Sci.

[17] Yan C, Boyd DD (2007). Regulation of matrix metalloproteinase gene expression.. J Cell Physiol.

[18] Price SJ, Greaves DR, Watkins H (2001). Identification of novel, functional genetic variants in the human matrix metalloproteinase-2 gene: role of Sp1 in allele-specific transcriptional regulation.. J Biol Chem.

[19] Yu C, Zhou Y, Miao X, Xiong P, Tan W, Lin D (2004). Functional haplotypes in the promoter of matrix metalloproteinase-2 predict risk of the occurrence and metastasis of esophageal cancer.. Cancer Res.

[20] Wojciechowski R, Bailey-Wilson JE, Stambolian D (2010). Association of matrix metalloproteinase gene polymorphisms with refractive error in Amish and Ashkenazi families.. Invest Ophthalmol Vis Sci.

[21] Nakanishi H, Hayashi H, Yamada R, Yamashiro K, Nakata I, Shimada N, Ohno-Matsui K, Mochizuki M, Ozaki M, Yoshitake S, Kuriyama S, Saito M, Iida T, Matsuo K, Matsuda F, Yoshimura N (2010). Single-nucleotide polymorphisms in the promoter region of matrix metalloproteinase-1, −2, and −3 in Japanese with high myopia.. Invest Ophthalmol Vis Sci.

[22] Leung KH, Yiu WC, Yap MK, Ng PW, Fung WY, Sham PC, Yip SP (2011). Systematic investigation of the relationship between high myopia and polymorphisms of the MMP2, TIMP2, and TIMP3 genes by a DNA pooling approach.. Invest Ophthalmol Vis Sci.

[23] Dupont WD, Plummer WD (1990). Power and Sample Size Calculations: A Review and Computer Program.. Control Clin Trials.

[24] Wojciechowski R, Moy C, Ciner E, Ibay G, Reider L, Bailey-Wilson JE, Stambolian D (2006). Genomewide scan in Ashkenazi Jewish families demonstrates evidence of linkage of ocular refraction to a QTL on chromosome 1p36.. Hum Genet.

[25] Paluru PC, Nallasamy S, Devoto M, Rappaport EF, Young TL (2005). Identification of a novel locus on 2q for autosomal dominant high-grade myopia.. Invest Ophthalmol Vis Sci.

[26] Hammond CJ, Andrew T, Mak YT, Spector TD (2004). A susceptibility locus for myopia in the normal population is linked to the PAX6 gene region on chromosome 11: a genomewide scan of dizygotic twins.. Am J Hum Genet.

[27] Zhang Q, Guo X, Xiao X, Jia X, Li S, Hejtmancik JF (2005). A new locus for autosomal dominant high myopia maps to 4q22-q27 between D4S1578 and D4S1612.. Mol Vis.

[28] Li YJ, Guggenheim JA, Bulusu A, Metlapally R, Abbott D, Malecaze F, Calvas P, Rosenberg T, Paget S, Creer RC, Kirov G, Owen MJ, Zhao B, White T, Mackey DA, Young TL (2009). An international collaborative family-based whole-genome linkage scan for high-grade myopia.. Invest Ophthalmol Vis Sci.

[29] Lam CY, Tam PO, Fan DS, Fan BJ, Wang DY, Lee CW, Pang CP, Lam DS (2008). A genome-wide scan maps a novel high myopia locus to 5p15.. Invest Ophthalmol Vis Sci.

[30] Paget S, Julia S, Vitezica ZG, Soler V, Malecaze F, Calvas P (2008). Linkage analysis of high myopia susceptibility locus in 26 families.. Mol Vis.

[31] Ciner E, Wojciechowski R, Ibay G, Bailey-Wilson JE, Stambolian D (2008). Genomewide scan of ocular refraction in African-American families shows significant linkage to chromosome 7p15.. Genet Epidemiol.

[32] Nallasamy S, Paluru PC, Devoto M, Wasserman NF, Zhou J, Young TL (2007). Genetic linkage study of high-grade myopia in a Hutterite population from South Dakota.. Mol Vis.

[33] Young TL, Ronan SM, Alvear AB, Wildenberg SC, Oetting WS, Atwood LD, Wilkin DJ, King RA (1998). A second locus for familial high myopia maps to chromosome 12q.. Am J Hum Genet.

[34] Yang Z, Xiao X, Li S, Zhang Q (2009). Clinical and linkage study on a consanguineous Chinese family with autosomal recessive high myopia.. Mol Vis.

[35] Paluru P, Ronan SM, Heon E, Devoto M, Wildenberg SC, Scavello G, Holleschau A, Makitie O, Cole WG, King RA, Young TL (2003). New locus for autosomal dominant high myopia maps to the long arm of chromosome 17.. Invest Ophthalmol Vis Sci.

[36] Young TL, Ronan SM, Drahozal LA, Wildenberg SC, Alvear AB, Oetting WS, Atwood LD, Wilkin DJ, King RA (1998). Evidence that a locus for familial high myopia maps to chromosome 18p.. Am J Hum Genet.

[37] Young TL, Atwood LD, Ronan SM, Dewan AT, Alvear AB, Peterson J, Holleschau A, King RA (2001). Further refinement of the MYP2 locus for autosomal dominant high myopia by linkage disequilibrium analysis.. Ophthalmic Genet.

[38] Stambolian D, Ibay G, Reider L, Dana D, Moy C, Schlifka M, Holmes T, Ciner E, Bailey-Wilson JE (2004). Genomewide linkage scan for myopia susceptibility loci among Ashkenazi Jewish families shows evidence of linkage on chromosome 22q12.. Am J Hum Genet.

[39] Klein AP, Duggal P, Lee KE, Klein R, Bailey-Wilson JE, Klein BE (2007). Confirmation of linkage to ocular refraction on chromosome 22q and identification of a novel linkage region on 1q.. Arch Ophthalmol.

[40] Zhang Q, Guo X, Xiao X, Jia X, Li S, Hejtmancik JF (2006). Novel locus for X linked recessive high myopia maps to Xq23-q25 but outside MYP1.. J Med Genet.

[41] Zhang Q, Li S, Xiao X, Jia X, Guo X (2007). Confirmation of a genetic locus for X-linked recessive high myopia outside MYP1.. J Hum Genet.

[42] Bartsocas CS, Kastrantas AD (1981). X-linked form of myopia.. Hum Hered.

[43] Haim M, Fledelius HC (1988). Skarsholm. X-linked myopia in Danish family.. Acta Ophthalmol (Copenh).

[44] Cirulli ETGD (2010). Uncovering the roles of rare variants in common disease through whole-genome sequencing.. Nat Rev Genet.

